# A Retroviral CRISPR-Cas9 System for Cellular Autism-Associated Phenotype Discovery in Developing Neurons

**DOI:** 10.1038/srep25611

**Published:** 2016-05-10

**Authors:** Michael R. Williams, Catherine J. Fricano-Kugler, Stephanie A. Getz, Patrick D. Skelton, Jeonghoon Lee, Christian P. Rizzuto, Joseph S. Geller, Meijie Li, Bryan W. Luikart

**Affiliations:** 1 Department of Physiology and Neurobiology, Geisel School of Medicine at Dartmouth College, USA

## Abstract

Retroviruses expressing a fluorescent protein, Cas9, and a small guide RNA are used to mimic nonsense *PTEN* mutations from autism patients in developing mouse neurons. We compare the cellular phenotype elicited by CRISPR-Cas9 to those elicited using shRNA or Cre/Lox technologies and find that knockdown or knockout (KO) produced a corresponding moderate or severe neuronal hypertrophy in all cells. In contrast, the Cas9 approach produced missense and nonsense Pten mutations, resulting in a mix of KO-equivalent hypertrophic and wild type-like phenotypes. Importantly, despite this mixed phenotype, the neuronal hypertrophy resulting from Pten loss was evident on average in the population of manipulated cells. Having reproduced the known Pten KO phenotype using the CRISPR-Cas9 system we design viruses to target a gene that has recently been associated with autism, KATNAL2. Katnal2 deletion in the mouse results in decreased dendritic arborization of developing neurons. We conclude that retroviral implementation of the CRISPR-Cas9 system is an efficient system for cellular phenotype discovery in wild-type animals.

Recombinant retroviruses based on the Moloney Murine Leukemia Virus (MoMuLv) integrate their genome into a host cell during the natural breakdown of the nuclear envelope during mitosis^1^. Thus, retroviruses are used to birth-date and genetically manipulate granule neurons in the postnatal mouse brain *in vivo*^2^. This property makes retroviruses ideal to study genetic mechanisms of neurogenesis and neuronal development because the precise age of labeled neurons is known - allowing for targeted study of specific developmental stages. This is particularly relevant to neurodevelopment disability associated genes, where the phenotype of gene KO may vary depending on neuronal age. Retroviral delivery of shRNA has been used to knockdown endogenous gene expression in wild type mice, while expression of Cre recombinase can be used to KO gene expression in floxed mice^3^,^4^. Virally delivered shRNA can result in inefficient knockdown rendering subtle and difficult to quantitate cellular phenotypes^5^. Cre-mediated recombination of floxed alleles results in complete KO and robust cellular phenotypes^4^, however, it necessitates the generation of genetically-engineered mice and is therefore inefficient for discovering cellular phenotypes of novel genetic manipulations.

The Clustered Regularly Interspaced Palindromic Repeats (CRISPR)-Cas9 system has established a powerful tool to target genetic manipulations to specific loci in a variety of non-genetically engineered organisms^6^,^7^,^8^,^9^. As applied in the laboratory, the Cas9 system has two main components, the Cas9 endonuclease, and a chimeric single guide RNA (sgRNA) which contains both a short sequence homologous to the gene of interest and sequences necessary for Cas9 interaction^10^. Although various mutant forms of Cas9 and Cas9 fusion proteins have been generated to alter specificity or activity, the normal RNA-guided endonuclease activity of Cas9 produces double stranded DNA breaks^10^,^11^. There are two types of repairs made at these breaks, error-prone Non-Homologous End Joining repair (NHEJ) and relatively precise Homology Directed Repair (HDR), however the latter’s requirement of G2 phase associated protein expression essentially excludes HDR in post-mitotic cells such as neurons^12^. Because the rapid process of NHEJ repairs double stranded DNA breaks without using a homologous DNA template, the repair can cause insertion or deletion of base pairs (indels) into the target region. These indels may create nonsense mutations early in a protein coding sequence, producing catastrophic frame shift mutations and premature truncation of the protein, acting essentially as a gene KO^12^, but without requiring transgenic animals.

Adeno-Associated Viral (AAV) particles have been used for Cas9 mediated gene KO in neurons *in vivo*^13^; however, the large size of *Streptococcus pyogenes* Cas9 (spCas9) necessitated co-injection of an AAV expressing Cas9 with another AAV expressing GFP and a sgRNA to target the gene of interest. Due to the inability to infect every cell with both viruses in a targeted brain region, cells infected with only one AAV will not have KO of the targeted gene. One strategy to overcome this limitation has been to use shorter Cas variants from other bacterial species, such as *Staphylococcus aureus* (SaCas9)^14^. However, the scaffold and guide considerations can be limiting, and this alternate strategy did not include a fluorescent reporter of infection. Lentiviral systems can accommodate much larger inserts than AAVs and can successfully deliver sgRNAs as well as Cas9 and a fluorescent protein via a single viral particle^15^. Similar to HIV-1 based lentiviral vectors, MoMuLv retroviruses also tolerate large inserts. Further, specificity for dividing cells makes MoMuLv retroviruses an attractive system to for phenotype discovery during development. We therefore engineered retroviruses expressing a sgRNA targeting the gene Pten, a fluorophore, and Cas9. We then compared the morphological phenotypes elicited using Cre-lox, shRNA, and CRISPR-Cas9 mediated disruption of Pten function.

## Results

### Approximating Autism-Associated Pten Mutations by Retroviral Cas9 Expression

**Figure 1:**
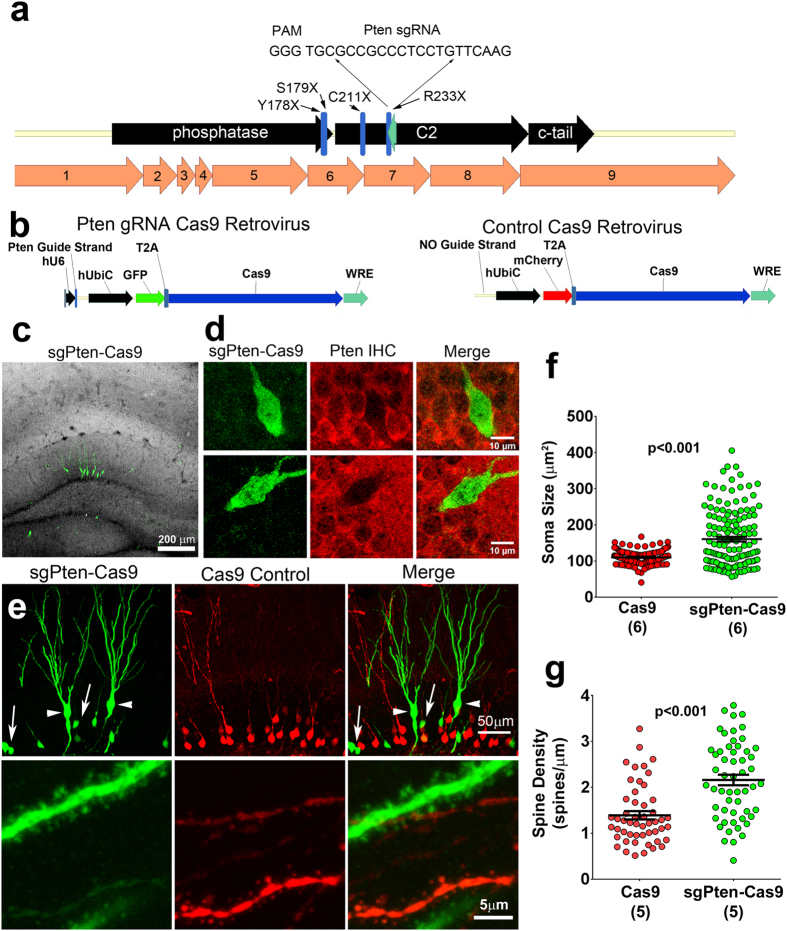
CRISPR-Cas9 Targeting of Pten Increases Neuronal Soma Size and Spine Density. (**a**) A schematic illustrating the position of the antisense Pten guide strand (sgRNA) within the coding sequence (black arrows) and exonic structure (numbered orange arrows) of mouse *Pten*. Nonsense mutations identified in ASD patients are labeled and indicated by blue lines. The Pten sgRNA was designed to approximate the R233X mutation. (**b**) Schematic depicting the Cas9 retroviral construct which uses the hU6 promoter to drive Pten sgRNA expression and the ubiquitin promoter to drive GFP and Cas9 expression, as well as a depiction of the control retroviral construct, mCherry-T2A-Cas9, which has no sgRNA cassette. (**c**) Retroviral injections were made into neonatal (P7) animals and histology performed 21-2 days post-injection. A maximum projection of low magnification optical sections reveals the gross pattern of sgPten-Cas9 infection is limited to granule neurons of the dentate gyrus (green), the general architecture is indicated by DAPI fluroesence (gray scale). (**d**) A single confocal plane reveals a loss of Pten immunoreactivity (red; Pten IHC) in two hypertrophic neurons infected with the GFP sgPten-Cas9 virus (green), when compared to surrounding uninfected granule neurons. (**e;** top) Low magnification of dentate granule neurons infected with sgPten-Cas9 retrovirus (green) depicts that there are hypertrophic neurons with morphology reminiscent of Pten knockout neurons (arrowheads) and there are some sgPten-Cas9 neurons (arrows), like those infected with the mCherry-T2A-Cas9 control (red), that appear normal. (**e**; bottom) High magnification images reveal increased thickness and higher spine densities of hypertrophic sgPten-Cas9 dendrites (green) in the same field of view as Cas9 control neurons (red). (**e**,**f**) Quantitative analysis indicates that the sgPten-Cas9 neurons have larger soma size (Cas9 control vs. sgPten-Cas9; p < 0.0001; n = 145 and 150 neurons in 6 animals) and dendritic spine density than Cas9 control neurons (Cas9 control vs. sgPten-Cas9, p < 0.001; n = 51 and 55 neurons in 5 animals).

Non-sense mutations resulting in stop codons between the phosphatase and C2 domains of Pten have been identified in patients with Cowden Syndrome or autism (Fig. 1a)^16^,^17^,^18^. To mimic this type of mutation we generated a sgRNA (sgPten) to target a Cas9 mediated double strand break in the mouse Pten gene near the corresponding site of the human R233X mutation (Fig. 1a). We cloned this sgPten and GFP-T2A-Cas9 into the pRubi (Retrovirus with internal ubiquitin promoter) backbone (sgPten-Cas9; Fig. 1b). To control for potential non-specific effects of Cas9 we also generated a mCherry-T2A-Cas9 control retrovirus with no sgRNA (Fig. 1b). We then co-injected these retroviruses in the hippocampus of postnatal day 7 (P7) mice and analyzed dentate gyrus granule neurons 21–24 days later (Fig. 1c). Cre-mediated KO, or shRNA mediated knockdown of Pten is known to produce hypertrophy in hippocampal granule cells^4^,^19^,^20^,^21^. Here, sgPten-Cas9 cells with morphology resembling Pten KO cells displayed no immunoreactivity with an antibody directed against the C2 domain of Pten (Fig. 1d). In our previous studies, this antibody has been confirmed to give specific Pten signal through the loss of immunoreactivity accompanying Pten knockdown or KO and by the increase of immunoreactivity accomplished by Pten overexpression^4^,^5^,^20^,^22^. Qualitatively, we noted that sgPten-Cas9 infected cells looked like a mixture of cells with wild-type and Pten KO morphologies (Fig. 1e).

Neuronal Pten KO has been associated with somatic hypertrophy and an increase in dendritic spine density^4^,^19^,^23^,^24^. Although there was a mixed population of phenotypically normal and hypertrophic soma, on average, sgPten-Cas9 neurons had an increased soma size (Fig. 1e top and Fig. 1f; control = 110.0 ± 1.50 μm^2^, sgPten-Cas9 = 160.5 ± 6.38 μm^2^, p < 0.001, from 145 and 150 neurons in 6 animals). We also found that sgPten-Cas9 increased dendritic spine density (Fig. 1e bottom and Fig. 1g; control = 1.39 ± 0.09 spines/μm, sgPten-Cas9 = 2.159 ± 0.11 spines/μm, p < 0.001, from 51 and 55 neurons in five animals).

### Pten Knockdown by Retroviral shRNA Expression

**Figure 2:**
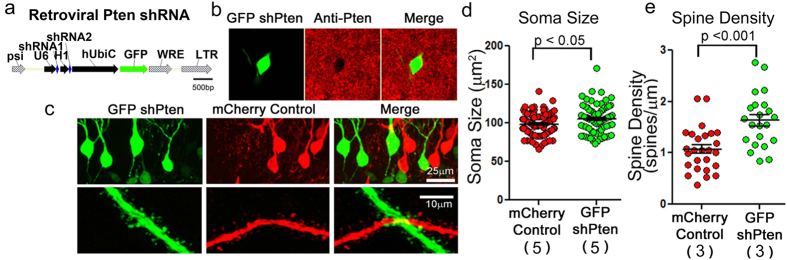
Retroviral Pten shRNA Knockdown Increases Soma Size and Spine Density. (**a**) Schematic depicting the retroviral construct which uses the H1 and U6 promoters to drive Pten shRNA expression and the Ubiquitin promoter to drive GFP expression. (**b**) A single confocal plane reveals a reduction in Pten immunoreactivity (red; Anti-Pten) in a neuron infected with the GFP shPten virus (green) when compared to the surrounding uninfected granule neurons in an adult animal *in vivo*. (**c**) Retroviral injection into neonatal (P7) animals and histology 21–24 days post-injection reveals that the GFP shPten infected neurons have larger soma sizes and higher spine densities. (**d**) The cross-sectional area of the soma is larger in the knockdown cells than in the control cells (mCherry control vs. GFP shPten; p < 0.05; n = 94 and 79 neurons from 6 and 5 animals, respectively). (**e**) There was an increase in spine density in the Pten knockdown neurons when compared to the control neurons (mCherry control vs, GFP shPten, p < 0.001; n = 26 neurons from 3 animals and n = 22 from 3 animals, respectively).

Like Cas9 based strategies, RNA interference through the expression of short hairpin RNA (shRNA) offers a technique to reduce the abundance of, i.e. to “knockdown”, a target protein without the use of genetically engineered animals. Unlike Cas9 strategies, shRNA strategies cannot mimic patient relevant gene mutations and rely on the continual production of the interfering RNA to deplete the target protein. We therefore sought to compare the phenotype caused by Cas9 and shRNA mediated Pten knockdown. To specifically knockdown Pten in newborn neurons of the dentate gyrus we developed a shRNA expressing retrovirus, using the pRubi retroviral backbone^21^ to drive GFP with the ubiquitin promoter and PTEN shRNAs with the H1 and U6 promoters (Fig. 2a). We have previously used lentiviruses to express this same validated shRNA to broadly target cells of the dentate gyrus and accomplish specific and efficacious (70–80%) knockdown of Pten transcript and protein *in vitro* and *in vivo*^5^,^20^. Analogous to the sgPten studies, C57BL/6 mice at P7 were co-injected with retroviral GFP shPten and a control retrovirus expressing mCherry (mCherry control; Fig. 2c). Between 21–24 days post injection (DPI), granule cells infected with shPten were found to have larger somas (Fig. 2c,d; GFP shPten, 106.7 ± 1.91 μm^2^; mCherry control, 97.59 ± 1.70 μm^2^; p < 0.05, t-test; n = 94 and 79 neurons from five animals, respectively) as well as higher spine densities (Fig. 2c,e; GFP shPten,1.64 ± 0.11 spines/μm; mCherry control, 1.07 ± 0.84 spines/μm; p < 0.001, t-test; n = 26 and 22 neurons from three animals, respectively).

### Pten Knockout by Retroviral Cre expression in Pten floxed Animals

**Figure 3:**
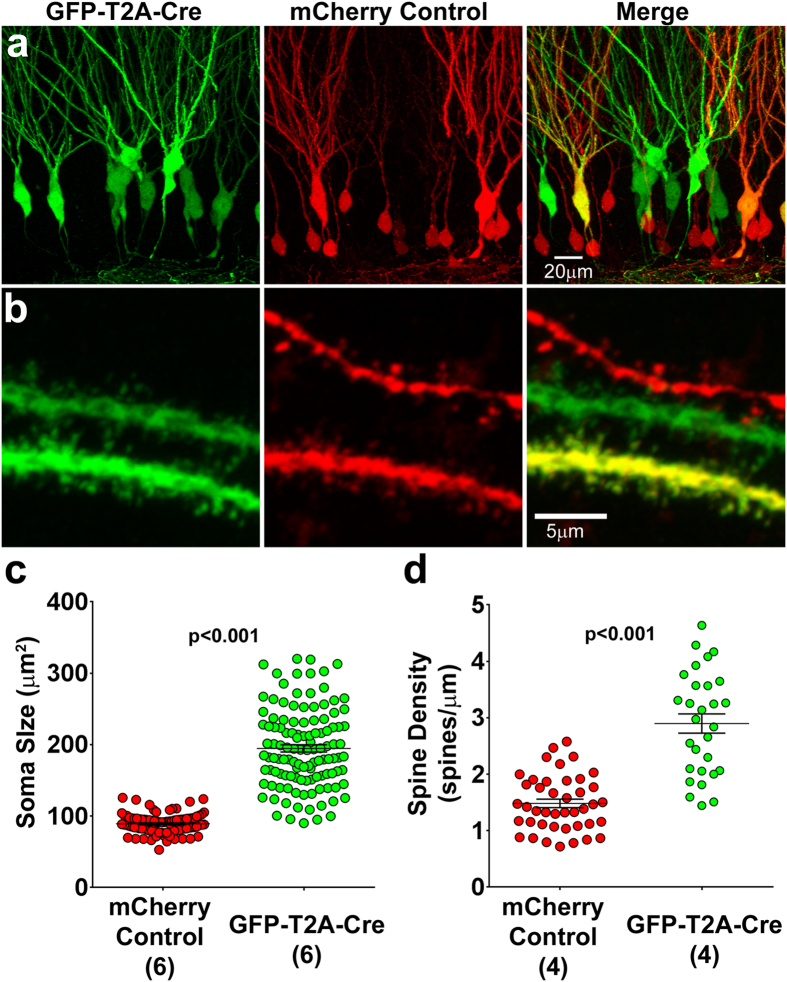
Retroviral Pten Cre-Lox Knockout Increases Soma Size and Spine Density. (**a**) Retroviral injection into P7 animals observed 21–24 days later reveals that the GFP-T2A-Cre infected neurons have larger soma sizes than the mCherry Control neurons. (**b**) High magnification images demonstrate that the GFP-T2A-Cre infected neurons have higher spine densities than neighboring mCherry Control neurons. (**c**) Quantitative analysis reveals that the cross sectional area of the soma is larger in the knockout cells than in the control cells (mCherry control vs. GFP-T2A-Cre; p ≤ 0.001; n = 94 and 108 neurons in 6 animals). (**d**) There was an increase in spine density in the PTEN knockout neurons when compared to the control neurons (mCherry control vs. GFP-T2A-Cre; p ≤ 0.001; n = 42 and 29 neurons in 4 animals).

Although relatively resource intensive, the generation of genetically engineered animals has tremendously facilitated the study of the genetic basis of development and disease. In animals wherein a genetic element of interest has been flanked by site directed recombinase targets such as Lox or FRT sites, the introduction of the corresponding recombinase (Cre or FLP) by breeding to another transgenic animal or by viral expression, results in irreversible gene knockout. We therefore sought to compare sgPten and shPten phenotypes to Cre mediated Pten KO. This was accomplished by co-injecting a GFP-T2A-Cre retrovirus with a mCherry expressing control retrovirus into Pten-floxed mice and performing histology between 21–24 DPI (Fig. 3)^4^. Pten KO neurons displayed a 2.18 fold increase in soma size compared to controls (Fig. 3a,c; control = 89.24 ± 1.42 μm^2^, Pten KO 194.8 ± 5.1; p < 0.001; from 94 control and 118 KO neurons in six animals). The Pten KO neurons had 1.96 fold increase in dendritic spine density (Fig. 3b,d; control = 1.48 ± 0.08 spines/μm, Pten KO = 2.90 ± 0.17 spines/μm; p < 0.001 from 42 control and 29 KO neurons in four animals).

### Quantitative Comparison of Cas9, -Cre, and shRNA Mediated Pten Manipulation

We observed that the Cre-mediated Pten KO produced the strongest and most consistent phenotype, the shPten phenotype was also homogenous but of lesser severity, and the sgPten yielded a population of mixed phenotypes (i.e. normal and hypertrophic neurons; Fig.1e,f). Supporting this observation, we found that the sgPten-Cas9 compared to the control condition had not only a greater mean soma size, but also a greater coefficient of variation (47.71% vs. 16.4% respectively). When using the D’Agostino & Pearson omnibus normality test and an alpha threshold of 0.01, the control group passed whereas the sgPten-Cas9 group did not. Collectively, these analyses suggested sgPten-Cas9 produced a mixed population of normal and hypertrophic neurons. In contrast, all other groups (i.e. control vs. shPten and control vs. Pten KO), passed the normality test.

**Figure 4:**
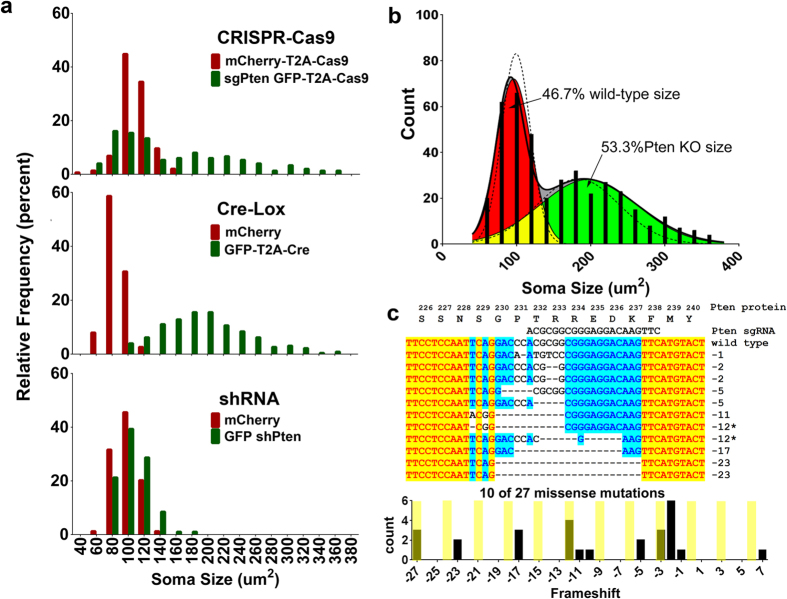
CRISPR-Cas9 Targeting of Pten Results in a Bi-Modal Distribution of Soma Size Indicative of Nonsense and Missense Mutations. (**a**) Frequency distribution of soma size for all neuronal populations examined in this study. The control populations (red) have a unimodal distribution. sgPten-Cas9 neurons resembled a mixture of wild-type and knockout cells. Soma sizes in Cre-positive Pten knockout neurons are much larger than in the mCherry Control neurons. Soma sizes in shPten knockdown neurons are slightly larger than in mCherry control neurons. (**b**) Distribution of soma size for sgPten-Cas9 neurons (black bars) was fit with a sum of two Gaussian distributions (black line). A single Gaussian fit for control and Cre-mediated knockout neurons (dotted lines) was used to constrain the overlapping boundaries of the bi-modal distribution to generate estimates of the percentage of neurons with wild-type (46.7%; red) and Pten knockout (53.5%; green) soma sizes within the mixed population of sgPten-Cas9 positive neurons. (**c**) Mouse Neuro-2a cells were infected with an sgPten-Cas9 lentivirus and a 232 bp region around the Pten sgRNA was PCR-cloned and sequenced. The Pten protein sequence is shown in frame with the sgRNA sequence, Pten nucleic acid sequence, and the sequence of 10 indels. The frequency distribution of all indels detected is shown in the histogram with in-frame missense mutations highlighted.

To characterize the relative phenotypic variability associated with sgPten we analyzed the frequency distribution of soma size for control neurons versus neurons expressing shPten, GFP-T2A-Cre or sgPten-Cas9 (Fig. 4a). We focused our analyses on soma size because it is established that there is a linear relationship between Pten expression levels and dentate gyrus granule neuron size^5^. Control, GFP-T2A-Cre and shPten neurons each displayed a unimodal distribution while the sgPten-Cas9 neurons had a bimodal distribution with peaks resembling wild-type and Cre-mediated Pten KO soma sizes. To estimate the percentage of cells resembling wild-type vs. Pten KO neurons among sgPten-Cas9 cells, we fit the distribution of 200 randomly selected control and 200 Cre-mediated Pten KO neurons with single Gaussian distributions and 400 sgPten-Cas9 neurons as the sum of two Gaussian distributions (Fig. 4b; Table 1). We then constrained the margin between the two peaks of the bimodal distribution with the fit lines for the control and Pten KO populations to estimate that 46.7% and 53.3% of the sgPten-Cas9 cells resembled wild-type and Pten KO cells, respectively (Fig. 4b). Therefore, we confirmed that the sgPten strategy produced a phenotypically heterogeneous population. However, this did not obscure the observation of neuronal hypertrophy on average at the population level.

### Analyses of sgPten Induced Mutations

We hypothesized that the generation of frame-preserving missense mutations versus nonsense mutations generated by NHEJ was responsible for the sgPten associated heterogeneity of soma sizes. To test this hypothesis we generated a lentivirus with an internal structure identical to the retrovirus used for the *in vivo* studies. We infected mouse Neuro-2a (N2A) cells with this virus, FACS isolated sgPten GFP-T2A-Cas9 positive cells and generated 232bp clones around the genome region targeted by the Pten sgRNA. Sequencing revealed 10 in-frame missense and 17 nonsense mutations (Fig. 4c). Nonsense mutations resulted in frameshifts between Gly230 and Arg233 with truncation at amino acid 255 for one base pair shifts and amino acid 242 for two base pair shifts. Thus we find that sgPten-Cas9 induced nonsense mutations result in a reasonable approximation of the R233X autism-associated mutation.

If sgPten induced NHEJ repair is a random process, one might expect 1/3 of clones to result in in-frame missense mutations. Supporting this, Sweich *et al*. used next generation sequencing to examine frameshifts in MeCP2 after CRISPR/Cas9 mediated NHEJ and found 34.1% missense and 65.9% nonsense mutations. If 2/3 of NHEJ repair results in nonsense mutation we would predict that 44.4% of neurons expressing the sgPten-Cas9 would be homozygous for a nonsense truncation, 44.4% heterozygous for a nonsense truncation and 11.1% homozygous for a missense mutation. From the analysis of soma sizes we estimated that 53.3% of sgPten-Cas9 neurons display soma sizes similar to Pten KO neurons. Cre-mediated deletion of Pten in heterozygous Pten floxed animals results in no detectable soma hypertrophy at 21–24 DPI (the mean and SEM of Pten Het KO and control soma areas was 102.3 ± 0.95 vs. 99.03 ± 1.49 microns^2^, from 266 vs. 192 neurons, respectively). Therefore it is unlikely that we could use morphology to distinguish heterozygous nonsense from homozygous missense mutations. Thus, our estimate of 53.3% homozygous nonsense mutations based on neuronal hypertrophy is greater than the 44.4% percent predicted nonsense mutations and likely includes loss-of-function mutations due to large (e.g., −27 bp, Fig. 4C) but frame-preserving mutations.

### Retroviral Cas9 Delivery for Phenotype Discovery of Autism-Linked Genes

**Figure 5:**
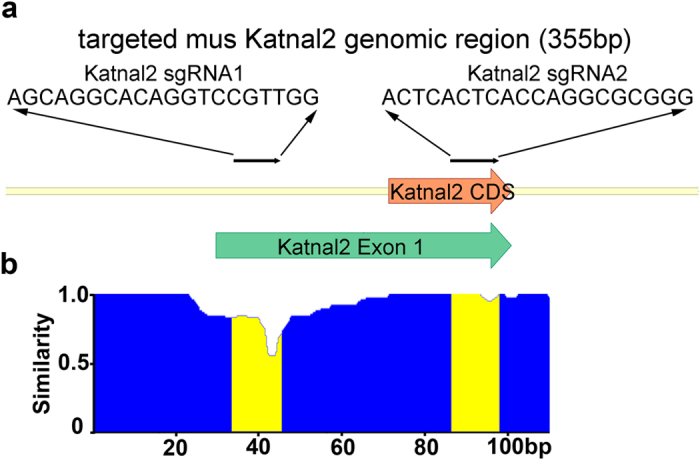
Retroviral CRISPR-Cas9 Targeting of Katnal2. (**a**) A schematic illustrating the position of the Katnal2 guide strands (black arrows, sgRNA) flanking the start codon in the coding region (orange arrow) of exon 1 (green arrow). (**b**) Neuro-2A cells were infected with a retrovirus expressing both Katnal2 sgRNAs. A plot of overall similarity for a sequence alignment between wild-type Katnal2 and 15 clones containing indels demonstrates a greater frequency of indel generation near sgRNA1. The sequence of each sgRNA is highlighted in yellow and aligns with the indicated sgRNAs in the schematic in (**a**).

Having found that retroviral delivery of sgRNAs targeting the established autism linked gene Pten was capable of producing a neuronal phenotype validated by KO studies, we sought to determine whether this system was capable of phenotype discovery for an understudied autism linked gene, Katanin P60 Subunit A-like 2 (Katnal2). Katnal2 is a presumptive microtubule-severing ATPase in which mutations have been associated with autism through whole-exome sequencing^25^,^26^,^27^. To disrupt mouse Katnal2 expression we designed a retrovirus expressing GFP, Cas9, and two sgRNAs flanking the start codon for the major predicted transcript variants 1, 2, and 4 (sgKatnal2 GFP-T2A-Cas9; Fig. 5a).

To confirm the efficacy of this strategy we infected N2A cells, isolated GFP positive cells with FACS, and cloned and sequenced the region of the Katnal2 gene targeted by the sgRNAs. In 15 sequenced clones we detected 14 clones with indels (mutations of base insertion and/or deletions) in the region near sgRNA1 and 2 indels proximal to sgRNA2 (Fig. 5b). We did not find any instance where the 111 bp region between the guide RNAs was excised. Given the position of sequenced indels this virus is likely to disrupt translation and/or result in catastrophic protein truncation. However, the commercial antibodies we tested for Katnal2 produced many non-specific bands in western blots and are therefore unreliable to evaluate protein expression.

**Figure 6:**
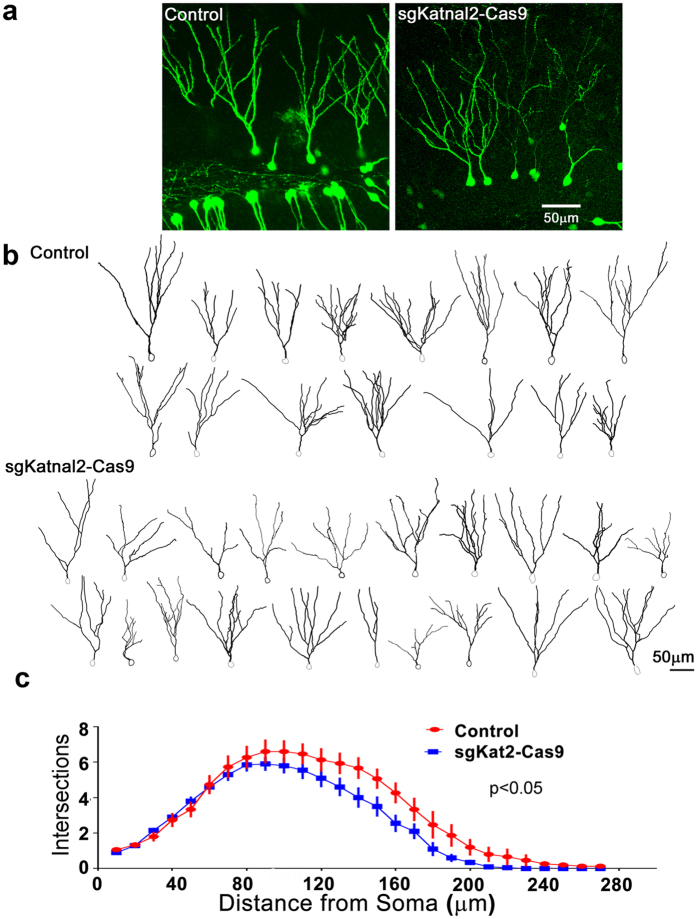
CRISPR-Cas9 Targeting of Katnal2 Results in Decreased Dendritic Growth. Neonatal (P7) mice were injected with retroviruses expressing GFP-alone (control) or a virus expressing both sgRNAs against Katnal2, GFP and Cas9. (**a**) Z-projections of 60 uM thick image stacks display the dendritic arbors of control and Katnal2 KO neurons. (**b**) Neurolucida reconstructions for all control and Katnal2 KO neurons demonstrate decreased dendritic complexity for Katnal2 KO neurons. (**c**) Scholl analysis shows a quantitative decrease in the number of branches intersecting the Scholl field at distal points.

We next injected the sgKatnal2 GFP-T2A-Cas9 retrovirus or a control retrovirus expressing GFP into the neonatal mouse dentate gyrus and evaluated granule cell morphology 21–24 DPI (Fig. 6a). Other Katanin family enzymes regulate dendritic and axonal morphology in zebrafish and drosophila^28^,^29^,^30^. We therefore first performed NeuroLucida reconstructions of GFP-labeled granule neurons (Fig. 6b) to measure parameters of dendritic arborization. Scholl analysis found that in the sgKatnal2-Cas9 condition, there was a reduced dendritic complexity as indicated by fewer branches at more distal distances from the soma (Fig. 6c; control = 15 reconstructions from 2 animals; sgKatnal2 GFP-T2A-Cas9 = 20 reconstructions from 2 animals; p < 0.05 2-way ANOVA with Bonferoni’s). In support of this analysis, we found that the sgKatnal2 GFP-T2A-Cas9 neurons had a reduced total number of branches (control = 8.80 ± 0.74, sgKatnal2-cas9 = 6.90 ± 0.42, p < 0.05). We also found that sgKatnal2 GFP-T2A-Cas9 neurons had a reduced total dendritic length (control = 957.70 ± 68.94 μm, sgKatnal2-cas9 = 756.20 ± 53.53 μm, p < 0.05). In these dendrite morphology analyses, we did not observe a difference in the coefficient of variability or the normality between Control and sgKatnal2 conditions. This may suggest that the inclusion of multiple guide RNAs against the same target gene reduces the variability of the emergent phenotype, that the phenotype is too subtle to parse distinct populations, or that, unlike Pten, heterozygous (rather than homozygous) nonsense mutations are sufficient to produce a discernible phenotype at this time point.

## Discussion

We find that retroviral implementation of the CRISPR-Cas9 system can be used to approximate nonsense Pten mutations identified in patients and that these nonsense mutations may cause neuronal hypertrophy equivalent to Pten KO. Despite the cell to cell variability apparently inherent to this strategy, we were able to clearly discern the established hypertrophic phenotype due to loss of Pten function across the cell population on average. Further, we demonstrated that retroviruses targeting Cas9 to the understudied autism candidate gene, Katnal2, disrupts normal dendritic branch formation in the developing mouse brain. These changes are consistent with hypotheses that disruption of normal neuronal development may lead to synaptic circuit dysfunction underlying the autism phenotype. Future studies into the cell autonomous physiological impact of Katnal2 KO, the impact of Katnal2 KO on neural circuit function, and the cellular mechanisms through which microtubule severing enzymes regulate neuronal growth and synapse formation are necessary.

The intrinsic specificity of retroviruses for dividing cells makes them suited to study genetic mechanisms regulating neuronal development. This is because viral expression marks the “birth” of a cell and the retroviral genome integrates into the host genome allowing for long term tracking of cell fate and physiology of infected cells. A single retroviral particle can deliver a fluorescent protein, sgRNAs, and Cas9, therefore fluorescence indicates the presence of Cas9 and the sgRNA with great fidelity. As a result, we have been able to demonstrate that retroviral strategies allow us to genetically manipulate, birth-date, and label cells in a manner sufficient for immunohistochemical, morphological, and physiological phenotype analysis. Because the Cas9-mediated genomic modification and the fluorescent marker gene are heritable this system will also be applicable for studies in cell division and differentiation. Further, the internal elements of the retroviral system are interchangeable with lentiviral systems to allow infection of non-dividing mature neurons or other post-mitotic/senescent cell populations.

One weakness of retroviral and lentiviral systems is that, unlike for AAVs, it is not currently possible to generate them with titers and diffusion characteristics allowing for infections of large brain regions. While retroviral gene knockdown has been used to probe gene/behavior linkages that are specific to neurogenesis^31^, except in those cases where behavioral change can be elicited by a small target region, lentiviral and retroviral-based techniques may be better suited for cell-biological rather than behavioral studies. Further, a caveat of retro/lenti -viral strategies is that although genomic integration enables labeling of cells for cell-fate studies, stable expression of Cas9 and sgRNAs could result in accumulation of off-target effects over time.

For researchers seeking to apply viral strategies for gene phenotype discovery, there should be a consideration of the relative importance of achieving specificity versus efficacy of disruption of the target. For the 20 bp sgRNAs of spCas9, algorithms have been established for calculating both the “on-target” (efficacy) and “off-target” (specificity) scores^32^,^33^. The online tool, Benchling, offers a user interface indicating both metrics on a scale of 1–100 with 100 indicating the highest efficacy and specificity, respectively. In this study, we designed the sgRNAs prior to the advent of the “on-target” score and, thus, selected sequences based on high “off-target” specificity scores. In our sequencing experiments, we found that the Katnal2 sgRNA2 was clearly less efficient than sgRNA1. It will be important in the future to determine whether algorithms predicting efficacy can facilitate better rational design of highly effective and specific sgRNAs^32^. Because not all guide RNAs are equally efficacious, the strategy of simply adding 2 guides for a target to produce efficient knockout is an oversimplification. It is therefore necessary to evaluate the efficacy of sgRNAs using surveyor assays or sequencing and to consider the use of emerging high-fidelity/enhanced specificity Cas9 variants.

Retro- and lentiviral Cas9 reagents will be usable in a wide variety of cell lines and tissues. We envision a major application for the retroviral system will be the study mechanisms of neurogenesis and for rapid cellular phenotype discovery. An array of CRISPR/Cas9 systems are available and users must consider what features are most salient to their scientific question. *In utero* electroporation can allow for rapid delivery of Cas9 constructs in the embryonic brain and is adaptable with piggyBAC systems to allow for lineage tracing^23^. Retroviral injection *in utero* may allow similar studies in tissue regions inaccessible to electroporation. AAV systems allow superior viral spread and are likely best suited for producing behavioral changes associated with gene modification in large brain regions^13^. For whole-animals studies, CRISPR/Cas9 modification of the oocyte genome is available^34^. The speed and flexibility of viral constructs and electroporation methods provide a complementary tool to identify genetic loci of sufficient biological relevance to justify the generation, validation, and utilization of transgenic animals for modeling genetic disorders.

## Methods

### Animals

Procedures were approved by Dartmouth Institutional Animal Care and Use Committee and conformed to federal, state, local, and Association for Assessment and Accreditation of Laboratory Animal Care standards. Mice from Jackson Laboratory were C57Bl/6J and B6.129S4-Pten <tm1hwu>/J homozygous (“Pten Floxed”) mice, also on a C57Bl/6J genetic background, of either gender. Animals were on a 12-hour light/dark cycle with chow and water provided *ad libitum*.

### Retrovirus and Lentivirus Constructs

Vectors and detailed vector maps are available on request, and through Addgene. Methods for the production of replication defective particles based on the Murine Maloney Leukemia Virus (“retrovirus”) using the pRubi transfer vector^20^ or based on the Human Immunodeficiency Virus using the FUGW transfer vector (“lentivirus”)^20^,^35^, have been previously described. Lentiviral particles have broad functional tropism due to an ability to transduce both mitotic and post-mitotic/senescent cells, while retroviral particles specifically transduce dividing cells, resulting in specific infection of newborn granule neurons when injected into the dentate gyrus.

The pRubi-fluorescent protein and pRubi-fluorescent protein-T2A-Cre constructs have been previously described^4^.

To create pRubi-GFP- shPten, we used an annealed oligo strategy with BbsI and BglII overhangs and the TTCAAGAGA loop sequence to place Human/Mouse Pten target sequences AGGTGAAG**G**TAT**G**TTCCTCCAA or AGGTGAAG**A**TAT**A**TTCCTCCAA into pCMV-based plasmids harboring the mouse U6 or human H1 RNA Pol III promoters, respectively. The U6 promoter and Pten shRNA was cloned into the retroviral pRubi backbone to generate pRubi U6 shPten using PacI/BstBI. The H1 promoter with Pten shRNA was cloned into pRubi U6 shPten to generate pRubi U6/H1 shPten using PacI. The final construct was sequence verified and named pRubi-GFP-shPten.

Retroviruses which birthdate, label, and utilize hSpCas9 for creating genetic lesions were created in stages to sequentially introduce hSpCas9 and the guide strand cassette. First, Not1 was used to linearize PX330^36^ (Addgene), from which hSpCas9 was amplified and EcoR1 flanked, by PCR. The PCR product was ligated into pGEM-T Easy and sequenced verified. By EcoR1 digestion, hSpCas9 was transferred an EcoR1 site downstream of a fluorescent protein-T2A sequence in pRubi-GFP and pRubi-mCherry, generating pRubi-GFP-T2A-hSpCas9 and pRubi-mCherry-T2A-hSpCas9.

The lentiviral constructs FU- GFP-T2A-hSpCas9 and FU-mCherry-T2A-hSpCas9 were generated by sequential digestion of FUGW, pRubi-GFP-T2A-hSpCas9, and pRubi-mCherry-T2A-hSpCas9 using Xho1 and then BstB1. The GFP/mCherry-T2A-Cas9 fragments were ligated into the FU backbone and sequence verified.

We next sought to facilitate the addition of the U6, guide strand, and RNA scaffold elements from PX330 into the viral plasmid backbones. We digested PX330 with PciI, the site for which precedes the U6 promoter. Into this PciI site, we ligated annealed oligos which regenerated the PciI site, and inserted downstream of it: CCTTAATTAACCTTCGAACC, encoding Pac1 and BstB1 sites. This modified plasmid was dubbed pXL, which also retained the original Pac1 site downstream of the U6 terminator.

Into BbsI digested pXL, we ligated annealed oligos for which the sense strand encoded a Bbs1 compatible overhang, G, and a guide sequence capable of targeting mouse Pten: AACTTGTCCTCCCGCCGCGT, Katnal2 guide1: AGCAGGCACAGGTCCGTTGG, and Katnal2 guide2: ACTCACTCACCAGGCGCGGG. The entire U6, guide strand, and RNA scaffold elements were digested out of pXL using BstB1 and Pac1, and ligated into pRubi- or FU- GFP-T2A-hSpCas9 digested with the same.

### Surgery

Stereotactic injection of viral particles into the brains of anesthetized mice was carried out as previously published^4^. Briefly, under isoflurane anesthesia, 2 μl injections of replication-defective retroviruses based on pRubi or FUGW were made into the dentate gyrus of each hemisphere at postnatal day 7, at a rate of at 0.3 μl/minute. The coordinates used for injection were (from lambda), y = +1.55, x = ±1.3, z = −2.3 to −2.0. At 21–24 days post-injection (DPI), animals were anesthetized by 2,2,2-tribromoethanol and transcardially perfused for immunohistochemistry using previously published methods^4^.

### Immunohistochemistry and Morphology Analyses

Methods for eGFP, mCherry, and Pten immunolabeling, and methods for neuronal soma size analysis, have been previously published^4^,^5^. Neuronal reconstructions were performed from confocal image stacks using NeuroLucida as previously described^4^. For dendritic spines, confocal z-stack image sets encompassed the thickness of individual infected granule neuron dendrites within the outer third of the molecular layer. Pten shRNA spine density was manually quantitated as previously described while the Cre and CRISPR-Cas9 manipulated spine densities used semi-automated analysis. For semi-automated analysis, image stacks were first deconvolved using ImageJ. By the ImageJ plugin Point Spread Function (PSF) Generator (Ecole Polytechnique Federale de Lausanne [EPFL]) and the Born and Wolf 3D Optical Model, an appropriate 8-bit PSF was created. The Richardson-Lucy algorithm was then used within the ImageJ plugin DeconvolutionLab (EPFL) with 5 iterations to generate deconvolved z-stacks. In NeuronStudio^37^, the deconvolved z-stack was opened as a maximum intensity z-projection. A region of interest was manually defined along a 10–15 micron representative span of dendrite in the middle of the image. NeuronStudio then automatically quantified the number of protrusions along the chosen segment. For both manual and semi-automated methods, spine density per dendrite was calculated as the number of protrusions per unit dendrite length. The term “spine” encompasses all dendritic protrusions in this context.

### Analysis of CRISPR-Cas9-induced genomic alterations

Mouse Neuro-2a (N2A, ATCC) cells were maintained on Matrigel basement matrix (reduced growth factor), in Iscove’s DMEM supplemented with fetal bovine serum, non-essential amino acids, penicillin and streptomycin, and glutamine. Parallel cultures were either left uninfected, or were transduced with FU-sgPten GFP-T2A-Cas9 lentiviral particles. Using the uninfected cells as a negative control, GFP-positive cells from the FU-sgPten GFP-T2A-Cas9 culture were enriched by fluorescence-activated cell sorting (FACS, Dartlab). Genomic DNA was isolated from the GFP-positive cells and subjected to PCR to amplify the region of the Pten gene (exon 5) corresponding to the sgPten target using the primer pair GGTCTGCCAGCTAAAGGTGA and CCAAAGGCTTTAAGCAAAAGG. Gel-purified PCR product was ligated into pGEM-T Easy, and the plasmid DNA isolated from transformants was sequenced utilizing a T7 primer. For Katnal2 the N2A cells were infected by 4 rounds of retrovirus addition to cell culture media, FACS isolation of GFP-positive cells and the genomic DNA was isolated with the following primer pair CTGTCGCCATAGCAACTGAA and ATTGCGCAGACTCTCTGGAT.

### Statistical Research Design

Unless otherwise indicated we used mixed-effect regression models with a random effect for each mouse to account for the fact that cells are clustered within mice, and therefore the cells from the same mouse are not statistically independent observations^38^. This mixed-effect model ensures that the confidence intervals and p-values accurately account for the varying number of cells from each mouse. The mixed-effect models were estimated using the xtmixed procedure in Stata (StataCorp. 2013. Stata Statistical Software: Release 13. College Station, TX: StataCorp LP). The pairwise comparisons were evaluated in Stata using the pwcompare command and the sch (Scheffe) option.

## Additional Information

**How to cite this article**: Williams, M. R. *et al*. A Retroviral CRISPR-Cas9 System for Cellular Autism-Associated Phenotype Discovery in Developing Neurons. *Sci. Rep*. **6**, 25611; doi: 10.1038/srep25611 (2016).

## Figures and Tables

**Table 1:** Best-fit Models Soma Sizes.

	Control	sgPten-Cas9		Pten KO
		Peak 1	Peak 2	
**Gaussian Best-Fit Values**				
Amplitude	83.10	62.22	28.49	28.50
Mean	99.04	90.71	190.8	183.0
SD	19.27	18.37	71.03	55.13
**Goodness of Fit**				
Robust Sum of Squares	91.17	6.130		8.067
RSDR	6.63 × 10^−5^	5.345		2.582
